# Development of Gastroretentive Floating Combination Tablets Containing Amoxicillin Trihydrate 500 mg and Levofloxacin 125 mg for Eradicating Resistant *Helicobacter pylori*

**DOI:** 10.3390/pharmaceutics16101242

**Published:** 2024-09-24

**Authors:** Da Hun Kim, Sa-Won Lee, Jun Hak Lee, Jin Woo Park, Sung Mo Park, Han-Joo Maeng, Tae-Sung Koo, Kwan Hyung Cho

**Affiliations:** 1 College of Pharmacy and Inje Institute of Pharmaceutical Sciences and Research, Inje University, 197 Inje-ro, Gimhae 50834, Republic of Korea; dahun990113@naver.com (D.H.K.); dlwnsgkr2341@gmail.com (J.H.L.); wlsdn4361@naver.com (J.W.P.); msk2241@gmail.com (S.M.P.); 2 Department of Pharmaceutical Engineering, Inje University, 197 Inje-ro, Gimhae 50834, Republic of Korea; lsw314@inje.ac.kr; 3 College of Pharmacy, Gachon University, 191 Hambakmoei-ro, Yeonsu-gu, Incheon 21936, Republic of Korea; hjmaeng@gachon.ac.kr; 4 Graduate School of New Drug Discovery and Development, Chungnam National University, 99 Daehak-ro, Yuseong-gu, Daejeon 34134, Republic of Korea; kootae@cnu.ac.kr

**Keywords:** gastroretentive floating combination tablets, sustained release, amoxicillin, levofloxacin, *Helicobacter pylori*, effervescent tablets

## Abstract

**Background/Objectives:** The aim of this work was to prepare and characterize gastroretentive floating combination tablets (GRCTs) containing 500 mg of amoxicillin trihydrate (AMX) and 125 mg of levofloxacin (LVX) that provide sustained drug release and stability at gastric pH levels for the eradication of resistant *Helicobacter pylori*. **Method:** GRCTs were prepared with low-density excipients and hydrophilic swellable polymers, including hydroxypropyl methylcellulose (HPMC) of various viscosities, polyethylene oxide (PEO), and carboxymethylcellulose (CMC), by the direct compression method. The prepared GRCTs were investigated and optimized in terms of pH stability, tablet hardness, floating lag time and total floating time, drug release rate, gel strength. **Results:** AMX and LVX in GRCT were stable at the HP eradication target pH above 4.0. The effervescent GRCT composition (AMX/LVX/HPMC [4000 cP]/CMC/microcrystalline cellulose/citric acid/sodium bicarbonate/calcium silicate/silicon dioxide/magnesium stearate = 500/125/50/50/125/40/60/30/10/10, *w*/*w*) yielded acceptable hardness (>6 kp), reduced floating lag time (<5 s), a long floating duration (>12 h), and sustained release rates of AMX and LVX (>90% until 12 h). This optimized GRCT had a gel strength of 107.33 ± 10.69 g and pH > 4.0, which maintained the tablets’ shape and AMX stability for 12 h. **Conclusions:** Collectively, the formulated effervescent GRCTs combining AMX and LVX represented a promising candidate dosage form for eradicating resistant *H. pylori*.

## 1. Introduction

The World Health Organization (WHO) identified *Helicobacter pylori* (HP) as one of the 12 most critical pathogens responsible for antibiotic resistance in 2017; however, the eradication of resistant HP remains an unmet challenge [1]. HP, a spiral-shaped Gram-negative bacterium that colonizes the gastrointestinal mucosa, is a common pathogen that infects more than 50% of the global population [2]. HP is a major cause of gastritis and peptic ulcers and is associated with 89% of non-cardiac gastric cancers and approximately 78% of all gastric cancers [3]. Because of these risks, the WHO has classified HP as a type 1 carcinogen [4]. Accordingly, there is an urgent need for advancing technology aimed at eradicating resistant HP.

According to global treatment protocols, a three-drug regimen comprising a proton-pump inhibitor (PPI) and two antibiotics is considered to be a standard HP eradication therapy [5]. PPIs maintain the stomach’s pH above 4 by inhibiting gastric acid secretion, which suppresses HP’s proliferation and enhances the efficacy of antibiotics against the bacterial cell division process. Thus, the use of PPIs and an increase in gastric pH are prerequisites for the action of antibiotics, maintaining the stability of antibiotics that are unstable in acid conditions [6,7]. Among the three-drug regimens comprising a PPI and two antibiotics, amoxicillin trihydrate (AMX) and clarithromycin are used as first-line therapy [8], while metronidazole and clarithromycin are used as alternative first-line therapies for patients who are allergic to AMX [8]. To be recognized as an effective bacteriostatic regimen, a success rate of at least 80% must be achieved in the intention-to-treat (ITT) analysis [9]. The ITT analysis, a method used in prospective randomized studies, includes all participants in the statistical analysis based on their original group assignment, regardless of whether they had previously received any treatment [10]. However, repeated HP bactericidal treatments have been associated with adverse events and antibiotic resistance worldwide. Currently, the failure rate of first-line microbicidal therapy is >20%, with an ITT of <80%; this result is difficult to recognize as bacteriostatic therapy [11]. To address this problem, second- and third-line treatments are being developed using combinations of novel antibiotics.

**Figure 1:**
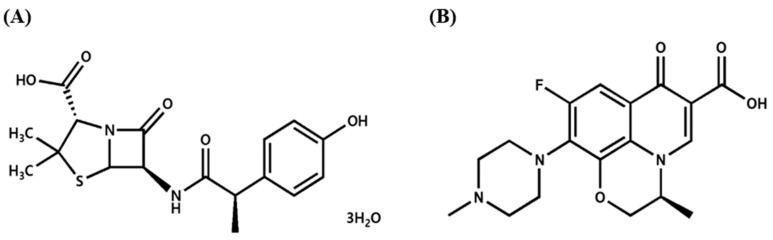
Chemical structures of (**A**) amoxicillin trihydrate and (**B**) levofloxacin.

One HP treatment regimen comprises a combination of AMX 1000 mg and levofloxacin (LVX) 250 mg twice a day with a PPI [12]. As shown in Figure 1, AMX possesses a beta-lactam ring structure and functions as an inhibitor of bacterial cell wall synthesis. This mechanism of action disrupts the integrity of the cell wall in both Gram-positive and Gram-negative bacteria, resulting in cell lysis and subsequent bacterial death [13]. LVX is a quinolone antibiotic used to treat symptoms caused by bacterial infections and functions by inhibiting bacterial DNA gyrase, which interferes with DNA replication and transcription and inhibits protein synthesis [14]. LVX was selected over other fluoroquinolones due to its superior efficacy against HP and its lower propensity for bacterial resistance. Moreover, LVX is highly absorbed in the gastrointestinal tract and exhibits excellent tissue penetration, making it an effective choice for treating HP infections. The combination of antibiotics with different mechanisms of action exerts a synergistic inhibitory effect on HP resistance.

HP microbicidal therapy often necessitates the continuous administration of multiple medications, which can result in suboptimal patient adherence. This issue can be addressed using drug combinations that amalgamate two or more active ingredients into a single formulation. In the present study, we developed a combination tablet containing AMX and LVX. This approach could enhance patient adherence by reducing the medication burden of multiple single-drug tablets [15]. The concurrent release of AMX and LVX from a single formulation would exert a local synergistic effect on the gastric mucosa, potentially increasing therapeutic efficacy. Additionally, the use of combination tablets may reduce healthcare costs for consumers and improve the manufacturing and distribution efficiencies of pharmaceutical companies, resulting in economic benefits [16].

Effective eradication of HP requires sustained localized action of antibiotics, such as AMX and LVX, on the gastric mucosa. However, conventional treatment methods result in a short gastric residence time of approximately 2–3 h, with most drugs absorbed systemically [17,18]. This limits their ability to maintain highly localized concentrations of antibiotics in the gastric mucosa. Gastroretentive drug delivery systems (GRDDSs) have been developed to address these limitations. GRDDSs can enhance both systemic and localized drug action by maintaining high drug concentrations in the stomach for extended periods and facilitating sustained release, thus optimizing absorption in the small intestine. GRDDSs can be categorized into expandable, floating, mucoadhesive, and high-density systems based on their gastric retention mechanisms [19]. Floating GRDDSs, which are low-density systems designed to remain buoyant in the stomach, allow prolonged gastric retention and controlled drug release. Floating GRDDSs are particularly advantageous because they are less influenced by factors such as age, race, and disease state and do not markedly impact gastrointestinal motility [20]. Considering these attributes, we developed a gastroretentive combination tablet (GRCT) incorporating both AMX and LVX, with the characteristics of a floating GRDDS. This formulation aims to enhance the therapeutic efficacy of the antibiotics by maximizing their local action in the stomach while ensuring optimal systemic absorption.

For targeted HP bactericidal therapy, GRCTs combining PPIs and antibiotics or a single antibiotic as a gastric suspension have been explored [21,22]. However, to the best of our knowledge, this is the first study to combine two antibiotics for HP bactericidal therapy, enabling a more localized action. Therefore, in this study, we developed GRCTs containing AMX and LVX using polymers and effervescent ingredients, and we evaluated various tablet properties and drug release properties to optimize the GRCT formulation.

## 2. Materials and Methods

### 2.1. Materials

AMX (purity > 98.0%) and LVX (purity > 98.0%) were purchased from Aladdin Industrial Corporation (Shanghai, China). Hydroxypropyl methylcellulose (HPMC; 100,000, 15,000, and 4000 cP) was kindly provided by Shin-Etsu Chemical Co., Ltd. (Tokyo, Japan). Sodium carboxymethylcellulose (CMC) was supplied by Ashland Inc. (Covington, OH, USA). Polyethylene oxide (PEO) was kindly provided by Colorcon Korea (Suwon, Republic of Korea). Aerosil 200 was obtained from EVONIK Industries (Hanau, Germany). All other chemicals were of reagent grade and used without further purification.

### 2.2. High-Performance Liquid Chromatography (HPLC) Conditions

The analysis of AMX and LVX in the samples was conducted using a 2695 HPLC system (Waters, Milford, MA, USA) equipped with a UV–vis detector (Waters 2487; Waters, Milford, MA, USA). All AMX and LVX analyses were conducted separately under the following conditions: AMX and LVX were analyzed using C18 (5 μm, 3.9 mm × 30 cm; Waters 2487, Waters, Milford, MA, USA) and C18 (5 μm, 4.6 mm × 15 cm; Osaka Soda, Osaka, Japan) columns, respectively. The mobile phase for AMX was a mixture of phosphate buffer (pH 5.0) and acetonitrile (39:1, *v*/*v*), while that for LVX was 0.87% pentanesulfonic acid in a mixture of phosphate buffer (pH 2.4) and acetonitrile (8:2, *v*/*v*). The HPLC analysis was performed at flow rates of 0.7 and 1.0 mL/min for AMX and LVX, respectively. The injected sample volume was 10 μL for both AMX and LVX. The UV detection was monitored at 230 nm for AMX and 294 nm for LVX. Data acquisition and processing were performed using the Waters LC Solution software (Empower 2.0).

### 2.3. pH Stability of AMX and LVX in Tablets

The pH stability of AMX and LVX was evaluated using buffer solutions of pH 1.2 (0.1 N HCl/0.034 M NaCl buffer), pH 4.0 (0.05 M acetate buffer), and pH 5.0 (prepared by titrating pH 4.0 buffer with 1 N NaOH). For testing pH stability, the tablets were prepared at a total mass of 1500 mg, containing 1 g of AMX, 250 mg of LVX, HPMC, CMC, and magnesium stearate, using the direct compression method. The tablet was placed in a drug dissolution tester (708-DS, Agilent Technologies Inc., Santa Clara, CA, USA) and tested in 900 mL of each buffer solution, with a paddle speed of 50 rpm, at 37 ± 0.5 °C. After 12 h, 5 mL samples were drawn and filtered through a 0.45 µm syringe filter (DISMIC^®^-13HP; ADVANTEC^®^, Tokyo, Japan). The filtrate was diluted with water and analyzed using a previously described HPLC method. The stability at each pH was evaluated using the percentage peak area of AMX and LVX (=peak area of each active ingredient × 100/total sum of all related peak areas) obtained from the chromatograms.

### 2.4. Preparation of GRCTs

GRCT formulations (F1–F11) were prepared using the direct compression method, with the tablet compositions detailed in Table 1. Each batch comprised 100 tablets, and the required quantity of each ingredient was weighed precisely for preparation. To ensure content uniformity, multiple manual mixing steps of 5 min were conducted, using plastic bags as containers. First, AMX, LVX, and Aerosil 200 were combined and mixed. Next, microcrystalline cellulose and each polymer (HPMC, PEO, or CMC) were added to the initial mixture. The subsequent step involved the incorporation of calcium silicate, citric acid, and sodium bicarbonate, followed by mixing. Finally, magnesium stearate was added as a lubricant, and the mixture was mixed thoroughly. The final blend was precisely weighed to 1 g in a weighing dish using an analytical balance (MS204; Mettler Toledo, Greifensee, Switzerland). The lower punch and die were filled with the mixture. After fitting the upper punch, the assembly was placed in a manual hydraulic press (SUG-10; SMART HYDRAULICS CO., LTD, Siheung-si, Republic of Korea) for direct powder compression [23]. For the tablet compression of F1–F8, the pressure was gradually increased to 30 bar using a pump, held for 5 s, and quickly released. The top punch was removed, and the bottom punch was raised to retrieve the GRCT. 

The effects of compression strength (compression pressure and dwelling time) on the physical and floating properties of the GRCTs were specifically evaluated using the F8 formulation. The compression pressures (30, 40, and 50 bar) and dwelling times (2 and 5 s) were varied as the primary process parameters. To prepare F9, F10, and F11, the compression pressure and dwelling time were set to 50 bar and 5 s, respectively.

### 2.5. Physical and Floating Properties of GRCTs

The physical properties of the prepared GRCTs, including weight, density, and hardness, were investigated. The weight and thickness of the prepared GRCTs were measured using an analytical balance (Mettler Toledo) and digital calipers (Vernier calipers, QST EXPRESS, Guangzhou, China), respectively [24]. Volume assessment of the GRCTs was conducted using a 50 mL graduated cylinder, initially filled with 10 mL of ethanol that did not cause disintegration or drug release of the tablet within 30 s. Each GRCT sample was subsequently immersed in a cylinder, and the resulting increase in volume was recorded. The density (D) of the GRCTs was determined using the previously measured weight (W) and volume (V) according to Equation (1):

D = W/V(1)

The hardness of the GRCTs, defined as their breaking force, was measured using a hardness tester (PTB 111E, PHARMA TEST, Hainburg, Germany).

To evaluate floating properties, both the lag time for complete floating and the total floating time of the GRCTs were measured by visual observation with a stopwatch using a 708-DS drug dissolution tester (Agilent Technologies Inc., Santa Clara, CA, USA) [25]. The tests were conducted under 900 mL of pH 4.0 buffer solution, with a paddle speed of 50 rpm, at 37 ± 0.5 °C. The floating and sinking behaviors of the GRCTs were observed and recorded visually. The criterion for floating was defined as GRCTs remaining on the surface of the buffer medium, whereas those below the surface were considered to have sunk [18]. All physical and flotation characterizations were performed in triplicate (*n* = 3) for each formulation, and the means and standard deviations were calculated.

### 2.6. Drug Release Test for GRCTs

The drug release test for the GRCTs was conducted using a drug dissolution tester (708-DS, Agilent Technologies Inc.) in 900 mL of pH 4.0 buffer solution (0.05 M), with a paddle speed of 50 rpm, at 37 ± 0.5 °C. For drug analysis, 5 mL aliquots were collected at predetermined intervals of 15, 30, 60, 180, 360, 540, and 720 min. Each collected sample was diluted 3-fold for AMX and 2-fold for LVX with distilled water. The diluted samples were filtered through a 0.45 µm membrane filter (DISMIC^®^-13HP, ADVANTEC^®^, Tokyo, Japan) and analyzed using HPLC. The HPLC conditions for AMX and LVX were consistent with those described earlier.

### 2.7. Measurement of Gel Strength and pH

For the selected GRCT formulations (F9–F11), gel properties were assessed in terms of gel strength and gel pH. Gel strength was evaluated by examining the GRCTs that remained in the drug release vessel after 6 h under the specified drug release test conditions, as described above. The wet GRCTs were uniformly distributed in the wells of 96-well plates and analyzed using a texture analyzer (TAXTplus, Stable Micro Systems, Godalming, UK). The pre-test speed was set at 0.1 mm/s, the trigger force was 0.5 g, and the test speed was 0.2 mm/s. Gel strength was defined as the peak positive force (g) during the passage of the plunger through the gel. The gel’s pH was measured by inserting a pH meter (SevenCompact S220, Mettler Toledo, Columbus, OH, USA) into the interior of the gel to determine the pH of the residual GRCT after 12 h under drug release test conditions. All gel property evaluations were performed in triplicate (*n* = 3), and the mean and standard deviation were calculated.

### 2.8. Drug Release Kinetics

The drug release data were fitted to kinetic models, including zero-order and first-order models, and the coefficient of determination (*r*^2^) was indicative of the best-fit model to describe the drug release kinetics [26].

## 3. Results and Discussion

### 3.1. pH Stability of AMX and LVX in Tablets

**Figure 2:**
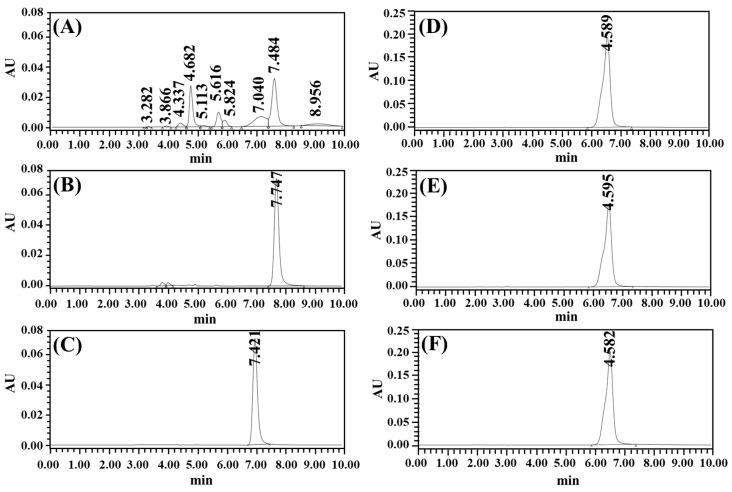
HPLC chromatograms of AMX and LVX in buffer solutions at 12 h from drug release testing of tablets: (**A**) AMX, pH 1.2; (**B**) AMX, pH 4.0; (**C**) AMX, pH 5.0; (**D**) LVX, pH 1.2; (**E**) LVX, pH 4.0; (**F**) LVX, pH 5.0.

The stability of AMX and LVX in the tablets was assessed in pH 1.2, pH 4.0, and pH 5.0 buffer solutions according to the drug release test. As shown in the chromatograms in Figure 2A, AMX exhibited significant instability at pH 1.2, as evidenced by a small AMX peak at a retention time of 7.48 min, along with several impurity peaks, indicating material degradation. This degradation was due to hydrolysis of the beta-lactam ring under acidic conditions, where protonation of the nitrogen atom by hydrogen ions led to ring opening, resulting in amines and their derivatives [27,28]. Furthermore, hydrogen ions interact with the carbonyl structure of the ring to generate degradation products, such as carbonyl acids and cyclic compounds [29]. In pH 4.0 and 5.0 buffers, the AMX peak was predominant, with slight or absent degradation peaks. There were only two small degradation peaks in the pH 4.0 buffer, indicating that AMX was substantially more stable at pH 4.0. The average peak area percentages of AMX at pH 1.2, 4.0, and pH 5.0 were 37.28, 95.88, and 99.71%, respectively. Conversely, LVX demonstrated no degradation peaks under any of the pH conditions tested, with an LVX peak area percentage of >99.0% at a retention time of ~4.59 min. LVX is highly stable over a broad pH range in the stomach. These results indicate that AMX and LVX were stable at pH > 4.0, which can be achieved by co-administering a PPI and is the therapeutic target pH for HP eradication [30]. All therapeutic guidelines for eradicating HP have adopted this strategy to maintain the stomach’s pH above 4.

### 3.2. Effects of Polymer Type on the Physical and Floating Properties of GRCTs

**Figure 3:**
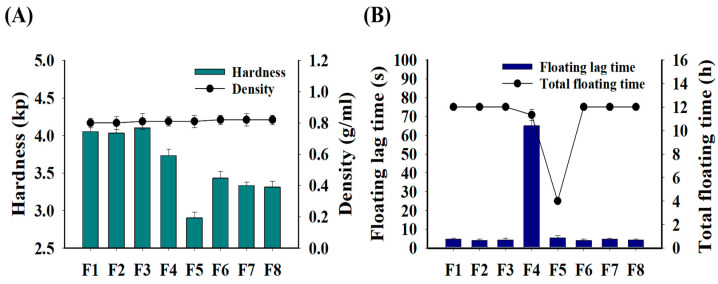
Physical and floating properties of F1–F8: (**A**) hardness and density; (**B**) floating lag time and total floating time.

The physical and floating properties of the GRCTs were evaluated based on the type of polymer used. The mean weight of all prepared GRCTs ranged between 997.47 and 998.47 mg/tablet, with a small relative standard deviation of <0.2%. This could be attributed to the manual tablet preparation using the direct compression method. Figure 3 presents the hardness and density of the GRCTs (F1–F8). The F6–F8 GRCTs, containing both CMC and HPMC, exhibited higher densities than F1–F3 without CMC. The intrinsic bulk density of CMC (0.520 g/mL) is higher than that of HPMC (0.341 g/mL) [31], leading to a smaller thickness and volume for GRCTs of the same mass. For all GRCTs, the densities were calculated from the measured weights and volumes and ranged between 0.80 and 0.82 g/mL, i.e., lower than the density of gastric juice (~1.004 g/mL), making them suitable for gastric floating [32]. The hardness of the GRCTs showed an inversely proportional correlation with their thickness, as a higher thickness indicated a higher volume and lower density of the tablet. Among all formulations, F1–F3, containing only HPMC, exhibited the highest hardness, with values of 3.9 ± 0.1, 4.0 ± 0.1, and 4.1 ± 0.1 kp, respectively. F4–F8, containing PEO or CMC alone or in combination with HPMC, had low mean hardness values of <3.80 kp, indicating that CMC and PEO reduced the hardness when compared to HPMC.

**Figure 4:**
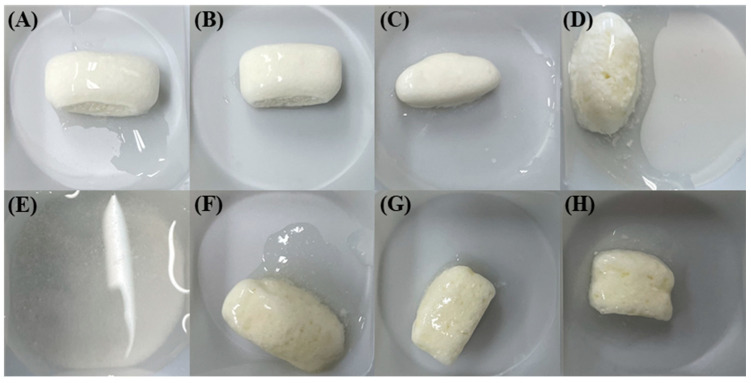
Visual observance of F1–F8 upon drug release testing at pH 4.0 at 12 h: (**A**) F1, (**B**) F2, (**C**) F3, (**D**) F4, (**E**) F5, (**F**) F6, (**G**) F7, and (**H**) F8.

The floating properties were evaluated based on the floating lag time and total floating time. Figure 3 presents the floating lag time and total floating time of the GRCTs (F1–F8). F1–F3 and F6–F8 demonstrated excellent floating lag times of <5 s and total floating times > 12 h. However, F4 had a relatively longer floating lag time (>1 min), and F5 was completely disintegrated at 4 h. The complete disintegration of F5 was attributed to the low viscosity of CMC (1500–2000 cP), which was insufficient to maintain the gel strength and shape of the GRCT over an extended period. Upon contact with water, the CMC in F5 absorbed water through capillary action and expanded, causing disintegration [33]. Figure 4 presents a visual illustration of the remaining GRCTs in the drug release vessels after evaluating their floating characteristics for up to 12 h. F1–F3 exhibited a greater degree of gel erosion with a smooth surface, along with a small residual amount remaining as the HPMC’s viscosity decreased [34]. Owing to the action of CMC, F6–F8 exhibited a greater degree of disintegration than F1–F3, along with the formation of some small holes and tears. F5, prepared with CMC as the polymer, was disintegrated completely, with no residual mass observed.

### 3.3. Effect of Polymer Type on the Drug Release Rate of GRCTs

**Figure 5:**
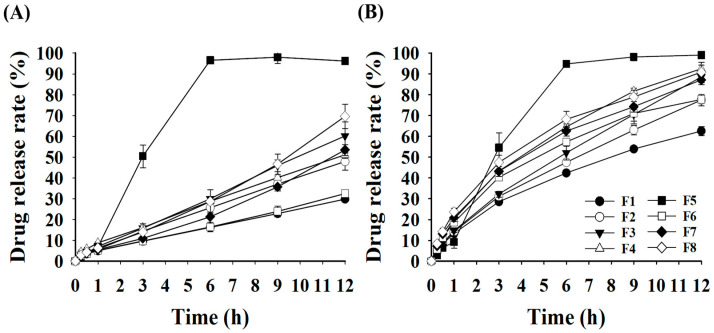
The drug release rates of F1–F8 at pH 4.0: (**A**) AMX and (**B**) LVX.

The drug release results of the GRCTs according to the polymer type are shown in Figure 5. For HP eradication therapy, PPIs are administered to maintain a stomach pH above 4.0. Therefore, pH 4.0 acetate buffer (0.05 M) was used as the drug release test medium for the formulated GRCTs. For all GRCTs except F5, AMX showed a lower drug release rate (<80% at 12 h) than LVX due to its higher dose strength (500 mg versus 125 mg) and greater interactions with HPMC and PEO [35]. Comparing the HPMC viscosities, the drug release rates of AMX and LVX at 12 h were in the order F3 (60.13% and 88.46%) > F2 (47.86% and 77.40%) > F1 (29.79% and 62.50%), which was in the opposite order of HPMC viscosity (F1 > F2 > F3). The higher HPMC viscosity resulted in the formation of a stronger gel layer and limited the diffusion of AMX and LVX molecules. At 12 h, F8, containing HPMC 4000 cP and CMC, exhibited the highest mean drug release rates for AMX and LVX, at 69.55 and 90.81%, respectively, compared with the other formulations, except for F5. In F8, the combined use of CMC and HPMC 4000 cP opened more release channels in the gelled tablets and increased the diffusion of drug molecules when compared with F3, formulated with HPMC 4000 cP alone [36]. At 12 h, F5 exhibited the highest drug release rates for AMX and LVX (>95%); however, this was attributed to the complete disintegration at ~4 h, indicating that the desired sustained release characteristics were not achieved. Therefore, F8 was selected for further investigations, although the drug release rate of AMX was incomplete and should be enhanced.

The drug release data for each formulation were fitted to zero-order or first-order kinetics to calculate the drug release rate constants. As shown in Table 2, AMX and LVX followed zero-order and first-order kinetics, respectively. All coefficients of determination (*r*^2^) for AMX and LVX in all of the formulations were more than 0.98, which means that the fitting showed good correlation within each kinetics model. In the comparison of the drug release rate constants for AMX, F8 showed the highest value (0.455) compared to the other formulations (0.196–0.345). In case of LVX, F8 also showed a high drug release rate constant of 0.717, which resulted in a high mean drug release rate of >90% at 12 h. The release rate constant increased as the viscosity of the HPMC decreased.

### 3.4. Effects of Compression Strength on the Physical and Floating Properties of GRCTs

**Figure 6:**
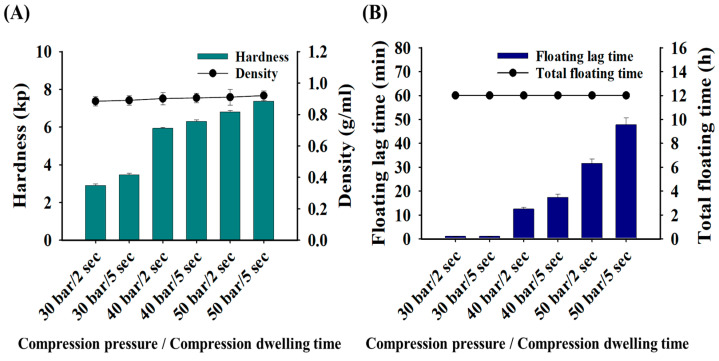
Physical and floating properties of F8 according to the compression strength (compression pressure and dwelling time): (**A**) hardness and density; (**B**) floating lag time and total floating time.

Using the F8 composition, we evaluated the effects of compressive strength (i.e., pressure and dwelling time) on the tablets’ hardness and floating properties. As observed in the above assessments, F8 exhibited a low hardness of 3–4 kp and an AMX drug release rate of <80% at 12 h, indicating the need to enhance the properties of this formulation. To address these issues, F8 was subjected to six different compression strengths to assess the changes in tablet density, hardness, floating lag time, and total floating time. As shown in Figure 6, the hardness of F8 increased with higher compression pressure and longer dwelling time. Under varying compression strengths, F8 maintained a low density of 0.91 g/mL and a high hardness of 7.4 kp at 50 bar/5 s. Despite achieving a total floating time exceeding 12 h with the compression strength, the floating lag time was 47 min, which is beyond the acceptable range of <1 min. The other three compression pressures (40 bar/2 s, 40 bar/5 s, and 50 bar/5 s) significantly increased the floating lag time to at least 10 min, with lower compression pressures (30 bar/2 s or 30 bar/5 s) resulting in a floating lag time of <1 min. Increasing the tablets’ compression pressure and dwelling time enhanced their hardness but adversely affected their floating properties, demonstrating the limitations of modifying compression strength alone to simultaneously improve both hardness and floating properties [37]. Therefore, to achieve a hardness of more than 6 kp while maintaining desirable floating properties, i.e., a short floating lag time and prolonged total floating time, we next examined the impact of incorporating effervescent agents, focusing on the enhancement of both properties.

### 3.5. Effects of Effervescent Agents on the Physical and Floating Properties of GRCTs

**Figure 7:**
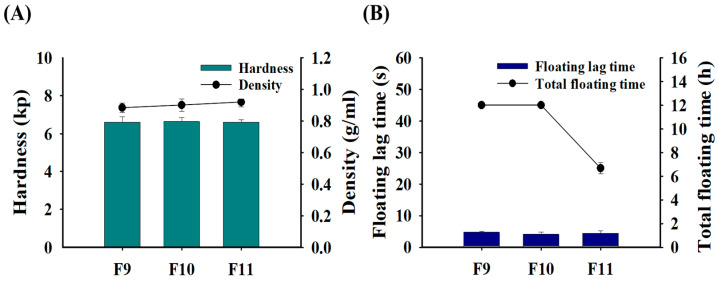
Physical and floating properties of F9–F11: (**A**) hardness and density; (**B**) floating lag time and total floating time.

The effects of increasing the percentage composition of citric acid and sodium bicarbonate, which are effervescent agents, on the physical and floating properties of the GRCTs were evaluated. Increasing the F8 hardness by changing the compression strength alone increased the floating lag time. Therefore, citric acid and sodium bicarbonate were added to the formulation to improve its hardness while maintaining good floating properties and increasing the drug release rate of AMX. As shown in Figure 7, the densities of F9–F11 ranged from 0.89 to 0.93 g/mL, all of which were sufficiently low to allow floating in the stomach [32]. The hardness of F9, F10, and F11 was 6.6 ± 0.3, 6.6 ± 0.2, and 6.6 ± 0.1 kp, respectively, approximately two-fold that of F8 (3.3 ± 0.1 kp) in Figure 3 [38]. Despite the improved hardness, F9–F11 exhibited excellent floating lag times (<5 s). In terms of total floating time, F9 and F10 exhibited a total floating time exceeding 12 h. Typically, effervescent tablets contain citric acid and sodium bicarbonate. Upon contact with water, these tablets generate carbon dioxide through an acid–base neutralization reaction and exhibit floating properties, with a sharp increase in the void volume in the tablet and a decrease in density. Thus, effervescent tablets can maintain floating ability even when their hardness and density increase due to strong compression [39]. However, F11 completely disintegrated at 6 h during the observation period, owing to the excessive amount of effervescent agent. Therefore, F9 and F10 were deemed to be suitable effervescent formulations in terms of hardness, floating lag time, and total floating time.

### 3.6. Effects of Effervescent Agents on the Drug Release Rate of GRCTs

**Figure 8:**
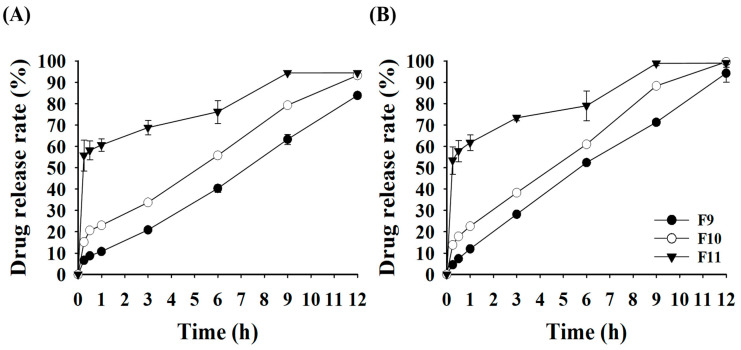
The drug release rates of F9–F11 at pH 4.0: (**A**) AMX and (**B**) LVX.

The effects of effervescent agents, specifically citric acid and sodium bicarbonate, on the drug release of the GRCTs were evaluated. To assess the drug release of the GRCTs (F9, F10, and F11), we employed acetate buffer (pH 4.0), which simulates the stomach pH environment in the presence of PPIs. As shown in Figure 8, both the initial and final drug release rates increased in proportion to the amount of the effervescent agent used. For F11, which contained the highest percentage of effervescent agents (15%), the final drug release rates of AMX and LVX at 12 h were 94.40 and 99.00%, respectively. However, owing to rapid initial disintegration, a substantial amount of the drug was dissolved within 1 h (60.63 and 61.71% of AMX and LVX, respectively). Complete disintegration occurred within 6 h, resulting in no remaining floating mass for 12 h, indicating a lack of sustained release. The drug release rate of F10, which comprised 10% effervescent agents, was higher than that of F8. The drug release rate of AMX increased from 6.35 to 22.99% at 1 h and from 69.55 to 93.22% at 12 h. For LVX, the drug release rate increased from 22.55 to 23.67% at 1 h and from 93.22 to 99.54% at 12 h. This confirmed that the effervescent agents in F10 enhanced the drug release rates of AMX and LVX from the GRCTs [40]. Both AMX and LVX demonstrated sustained release for 12 h.

Among the formulations containing effervescent agents (F9 and F10), the drug release rate constants for AMX and LVX followed zero-order kinetics and first-order kinetics, respectively. In particular, the drug release rate constants of AMX for F9 and F10 were 0.554 and 0.592, respectively, which were higher compared to that of F8 (0.455) shown in Table 2. For LVX, F9 (0.845) and F10 (0.831) showed similar drug release rate constants to F8 (0.847). For F11, due to the large initial burst release within 30 min, the drug release rate constant could not be determined, as it did not fit either of the drug release kinetics models.

**Figure 9:**
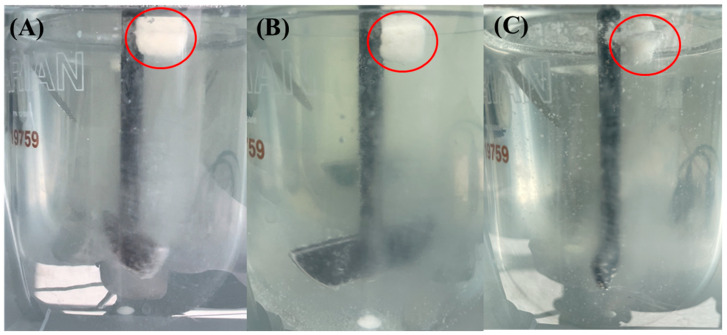
Visual observance of F10 upon drug release testing at pH 4.0: (**A**) 0 h, (**B**) 6 h, and (**C**) 12 h. Red circles indicate the remaining GRCT 10.

**Figure 10:**
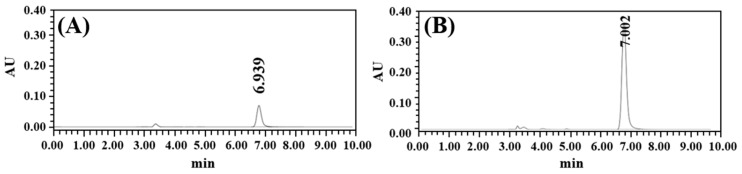
Chromatogram of AMX analysis from the drug release sample of F10 at pH 4.0: (**A**) 15 min and (**B**) 12 h.

As indicated by the red circle in Figure 9, optimized F10 maintained an adequate floating mass for 12 h. Moreover, as depicted in Figure 10, the peak area % of AMX in the chromatogram at 12 h was 96.77%, indicating its chemical stability in the drug release medium without significant hydrolysis. Optimizing the amount of effervescent agents could effectively improve the physical strength and floating properties, as well as enhancing the sustained release characteristics of the formulation.

### 3.7. Gel pH and Strength of GRCTs

**Figure 11:**
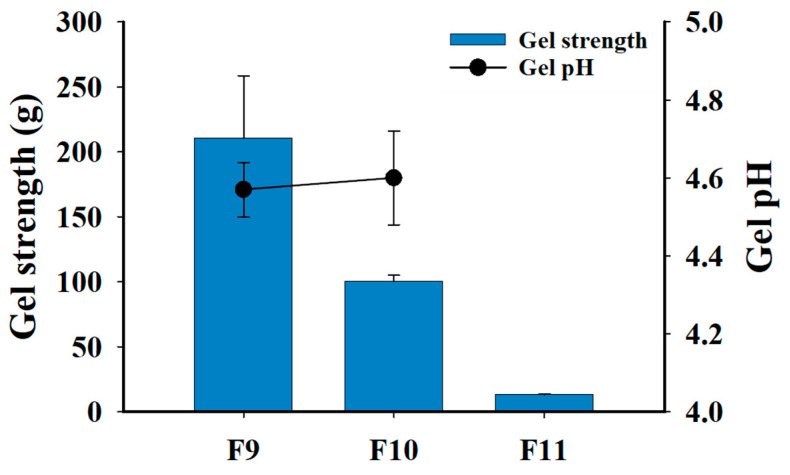
Gel strength and pH of F9–F11 upon drug release testing at pH 4.0 at 12 h.

The gel strength and pH of the GRCTs (F9–F11) are shown in Figure 11. After 6 h of drug release testing, the gel strengths of F9, F10, and F11 were 210.6 ± 47.9, 107.3 ± 10.7, and 13.2 ± 0.7 g, respectively. These results indicate that increasing the amount of the effervescent agent reduced the gel strength of each formulation [41]. This reduction in gel strength could be attributed to the increased void volume within the tablets owing to gas production, which decreased the density of the gelling polymer and weakened the gelling layer [42]. When the gel strength is too weak, the tablet disintegrates quickly, and gastric emptying is promoted. Moreover, sustained release will be impossible to achieve. Therefore, maintaining suitable gel strength by modifying the amount of effervescent agent is critical for the gastric retention and sustained release. The optimized F10 exhibited sufficient gel strength (>100 g) to maintain the gel structure in the irregular environment of the stomach for up to 12 h. At 12 h, the gel pH of F9 and F10 was 4.4 ± 0.1 and 4.6 ± 0.1, respectively. This pH range (˃4.0) was achieved due to the combined pH characteristics of the drug release test medium and the tablet ingredients, ensuring the chemical stability of the AMX and LVX. For F11, the gel pH could not be measured owing to the complete disintegration at 6 h. The optimized F10 maintained gel strength and a chemically stable gel pH above 4.0, along with sustained drug release rates and floating properties for 12 h.

## 4. Conclusions

In this study, GRCTs containing 500 mg of AMX and 125 mg of LVX were formulated and developed to eradicate resistant HP. The GRCT formulations incorporated HPMC and CMC with effervescent agents, i.e., citric acid and sodium bicarbonate, to enhance the tablets’ properties and drug release profiles. Stability tests confirmed that AMX and LVX remained stable at pH > 4.0, which is essential for effective HP therapy. The GRCT formulations (F9, F10, and F11) revealed that increasing the percentage of effervescent agents improved the drug release rates. Specifically, F10, which contained 10% effervescent components, demonstrated the most balanced performance between enhanced sustained and high drug release rates, along with suitable floating properties for 12 h. This formulation maintained a gel strength of >100 g at 6 h and pH 4.6 at 12 h, ensuring its stability and structural integrity in the gastric environment. Overall, the optimized GRCT (F10) is a promising dosage form for eradicating resistant HP, with prolonged gastric retention, sustained drug release, and enhanced therapeutic efficacy by maximizing the local antibiotic action in the stomach.

## Figures and Tables

**Table 1:** The formulation compositions for GRCTs.

Ingredients (mg/tablet)	F1	F2	F3	F4	F5	F6	F7	F8	F9	F10	F11
AMX	500	500	500	500	500	500	500	500	500	500	500
LVX	125	125	125	125	125	125	125	125	125	125	125
HPMC (100,000 cp)	100	-	-	-	-	50	-	-	-	-	-
HPMC (15,000 cP)	-	100	-	-	-	-	50	-	-	-	-
HPMC (4000 cP)	-	-	100	-	-	-	-	50	50	50	50
PEO	-	-	-	100	-	-	-	-	-	-	-
CMC	-	-	-	-	100	50	50	50	50	50	50
Citric acid	-	-	-	-	-	-	-	-	20	40	60
Sodium bicarbonate	-	-	-	-	-	-	-	-	30	60	90
Microcrystalline cellulose	225	225	225	225	225	225	225	225	175	125	75
Calcium silicate	30	30	30	30	30	30	30	30	30	30	30
Aerosil 200	10	10	10	10	10	10	10	10	10	10	10
Magnesium stearate	10	10	10	10	10	10	10	10	10	10	10
Total weight	1000	1000	1000	1000	1000	1000	1000	1000	1000	1000	1000

**Table 2:** Drug release rate constants (zero-order rate constant (*K*_0_) and first-order rate constant (*K*_1_)) and coefficients of determination (*r*^2^) of AMX and LVX for F1–F8.

Formulation	AMX		LVX	
	*K*_0_	*r* ^2^	*K*_1_	*r* ^2^
F1	0.196	0.994	0.675	0.998
F2	0.326	0.998	0.698	1.000
F3	0.409	0.999	0.719	0.999
F4	0.340	0.994	0.730	0.996
F5	N/A *		N/A *	
F6	0.211	0.995	0.707	0.993
F7	0.345	0.985	0.708	0.991
F8	0.455	0.988	0.717	0.985

* Not available.

## Data Availability

The datasets generated or analyzed during the current study are available from the corresponding author upon reasonable request.
